# Determining the Nutrient Content of Hydroponically-Cultivated Microgreens with Immersible Silicon Photonic Sensors: A Preliminary Feasibility Study

**DOI:** 10.3390/s23135937

**Published:** 2023-06-26

**Authors:** Aristi Christofi, Georgia Margariti, Alexandros Salapatas, George Papageorgiou, Panagiotis Zervas, Pythagoras Karampiperis, Antonis Koukourikos, Petros A. Tarantilis, Eleftheria H. Kaparakou, Konstantinos Misiakos, Eleni Makarona

**Affiliations:** 1 Institute of Nanoscience and Nanotechnology, NCSR “Demokritos”, 153 41 Athens, Greece; aristi.christofi@gmail.com (A.C.); margaritigeo@gmail.com (G.M.); a.salapatas@inn.demokritos.gr (A.S.); g.papageorgiou@inn.demokritos.gr (G.P.); k.misiakos@inn.demokritos.gr (K.M.); 2 Department of Materials Science, University of Patras, 265 04 Rio, Greece; 3 SCiO P.C., 153 10 Agia Paraskevi, Greece; panagiotis@scio.systems (P.Z.); pythagoras@scio.systems (P.K.); antonis@scio.systems (A.K.); 4 Laboratory of Chemistry, Department of Food Science and Human Nutrition, School of Food and Nutritional Sciences, Agricultural University of Athens, 118 55 Athens, Greece; ptara@aua.gr (P.A.T.); elek@aua.gr (E.H.K.)

**Keywords:** optical biosensors, photonic circuits, real-time spectral analysis, microgreens, basil, total phenolic content, antioxidant activity, hydroponics, urban farming

## Abstract

Microgreens have gained attention for their exceptional culinary characteristics and high nutritional value. The present study focused on a novel approach for investigating the easy extraction of plant samples and the utilization of immersible silicon photonic sensors to determine, on the spot, the nutrient content of microgreens and their optimum time of harvest. For the first time, it was examined how these novel sensors can capture time-shifting spectra caused by the molecules’ dynamic adhesion onto the sensor surface. The experiment involved four types of microgreens (three types of basil and broccoli) grown in a do-it-yourself hydroponic installation. The sensors successfully distinguished between different plant types, showcasing their discriminative capabilities. To determine the optimum harvest time, this study compared the sensor data with results obtained through standard analytical methods. Specifically, the total phenolic content and antioxidant activity of two basil varieties were juxtaposed with the sensor data, and this study concluded that the ideal harvest time for basil microgreens was 14 days after planting. This finding highlights the potential of the immersible silicon photonic sensors for potentially replacing time-consuming analytical techniques. By concentrating on obtaining plant extracts, capturing time-shifting spectra, and assessing sensor reusability, this research paves the way for future advancements in urban farming.

## 1. Introduction

The United Nations (UN) predicts that the global population will reach 9.7 billion by 2050, with 70% residing in urban areas [1,2]. Consequently, about 80% of produced food is expected to be consumed in cities by 2050 [3], necessitating a drastic revision of food production methods worldwide. This urgency is further exacerbated by the triple threats of climate change, biodiversity loss, and pollution, which lead to the continuous loss of arable land. Urban agriculture, which is defined as “the production, processing, and distribution of food and other products by plants and/or livestock raised in and around cities to meet local needs” [4], has emerged as an appropriate solution to address the challenge of securing a sustainable global food production system and promoting circular economies within the growing number of megacities in all continents [3,4,5,6,7]. One of the fundamental components of urban agriculture is hydroponic farming, where plants are grown in water instead of soil. Hydroponic plants are grown in a nutrient-rich aqueous solution that provides support and nourishment. As such, hydroponic farms can be established in any urban location at a low cost for home-based solutions, regardless of soil or climate conditions. Hydroponics is a more sustainable method compared to traditional farming because it saves water, space, and energy. It uses up to 98% less water, occupies 99% less land than outdoor soil-based farms, and requires only 21% of food production energy. Hydroponic installations are easily deployable and accessible, making it possible to produce fresh vegetables even in households.

Microgreens represent a particular class of hydroponically cultivated products that are the seedlings of certain crop species; they are harvested at the first true leaf stage by cutting the seedlings 5–10 mm above the growing media surface. These tiny plants have recently gained recognition as high-value functional foods due to their remarkable nutritional properties [8]. In fact, microgreens are rich in various antioxidants and essential minerals, such as potassium, calcium, iron, and zinc. Recent studies have revealed that microgreens contain higher amounts of certain vitamins, sugars, and antioxidants than mature greens, including valuable carotenoids. Consuming microgreens has also been associated with numerous health benefits, such as a reduced risk of cardiovascular disease and increased protection against inflammatory processes [9,10,11,12,13,14]. As with all plants, several factors can affect their nutrient content, such as light conditions, water nutrients, and growth substrate materials [12,15,16,17]. Given the importance of microgreens for sustainable food production and associated health benefits, it is essential to find ways to rapidly and accurately assess the optimum time of harvest and how growth conditions should be controlled and varied. With a cultivation time of just 21 days and hydroponic installations envisioned to be scattered within urban settings (houses, offices, terraces, rooftops, communal gardens, etc.), traditional analytical methods relying on laborious plant extraction and laboratory equipment, such as liquid chromatography electrospray ionization quadrupole time-of-flight mass spectrometry (LC-ESI-QTOF/MS) [18] or metabolomics and transcriptomics analyses [19] with long turn-around times, may not be a viable solution for a quick assessment of the optimum harvesting time. On the other hand, sensor-based analyses are mostly used to monitor, control, and optimize cultivation conditions or crop health and growth rates in terms of physiological parameters (color of leaves, stem length) and not to assess the plant nutrient contents [20,21,22].

This study explores an alternative method for determining the nutrient content of microgreens by using hydroponically grown basil microgreens as a test case. In contrast to recent approaches that use light to determine the nutrient content of soil or plants through spectroscopic methods, such as FT-IR, TXRF, and UV/VIS [23,24,25,26,27], color analysis [28,29], or spectral imaging [30], the proposed approach uses “light” and spectroscopic analysis in a different way. It employs a novel type of immersible Si-based photonic sensor using Broad-band Mach–Zehnder Interferometry (BB-MZIs) as the principle of operation [31,32,33]. The two most important novelties behind this type of sensor make it stand out and render it suitable for this study, as presented in detail in [34] and as will be further analyzed in Section 2.2: (1) Contrary to “traditional” biosensors, they do not require any microfluidics, but can be directly immersed into the analytes. This feature alleviates the need for tubes and pumps. (2) The coupling of the sensor to the source and the recording medium is performed in a purely optomechanical way, alleviating the need for tedious alignment procedures and expensive optical components. These two features render the immersible photonic sensors ideal for creating compact and light-weight biosensing systems for on-the-spot determinations.

In this study, the sensors are immersed in basil pulps at the hydroponic installation site, and the output signal is a dynamically shifting spectrum. However, signal analysis is simplified by using Fast Fourier Transform (FFT) to record a single variable: the phase change over time due to biomolecular interactions [34]. The immersible sensors were originally designed as a rapid, portable, and cost-effective tool for biodiagnostics and food safety determinations, and they have been appropriately functionalized with biorecognition molecules for specific analytes in these contexts [34,35]. In this work, however, the BB-MZI sensing areas have not been modified with any biorecognition molecules, nor have they undergone any surface treatment. Any molecule that can adhere to the nitride upper surface will change the effective medium through which light is transmitted and the associated waveguide effective index of refraction. This will be manifested as a spectral shift in the output spectral content. Here, it is postulated that the various molecules of a plant extract can interact with the Si_3_N_4_ surface due to surface adhesion, with the notion that the gradient of the phase changes induced by the various contents and their relative concentration in a plant’s extract may be indicative of and correlated to its nutrient content.

This concept was first tested with various microgreen pulps in order to establish that the gradient of the phase changes differs from plant to plant and within plants of the same species. Subsequently, two types of basil microgreens were examined in 1-week intervals for a period of 3 weeks, and the results obtained from the immersible photonic sensors were compared to the total phenolic content and antioxidant activity, as determined by Folin–Ciocalteu, DPPH, and ABTS assays. This study showed that there is a correlation between the signals detected by the immersible photonic sensors and the total phenolic content of the microgreens. These findings suggest that the best time to harvest hydroponically grown microgreens is at 14 days (2 weeks after planting). While these results are preliminary and only serve as proof of concept, they indicate that these novel photonic sensors could potentially be used to determine the nutrient content of plants. This could lead to the development of a new, rapid, cost-effective, and efficient tool that can be used on-site by anyone to determine the optimal harvest time for hydroponically farmed plants.

## 2. Materials and Methods

This feasibility study was developed with the following underlying concept: to create an affordable and accessible hydroponic installation with personalized monitoring for use in any urban setting, even by non-professionals. All components must be off-the-shelf, affordable, and easily ordered online, while the installation can be assembled in a do-it-yourself (DIY) manner. The optical biosensing platform should be portable and autonomous, and the optical sensors should be reusable. The measuring process should be simple, cost-efficient, and performed in a room or office with easily accessible reagents.

### 2.1. Hydroponic Installation

**Figure 1:**
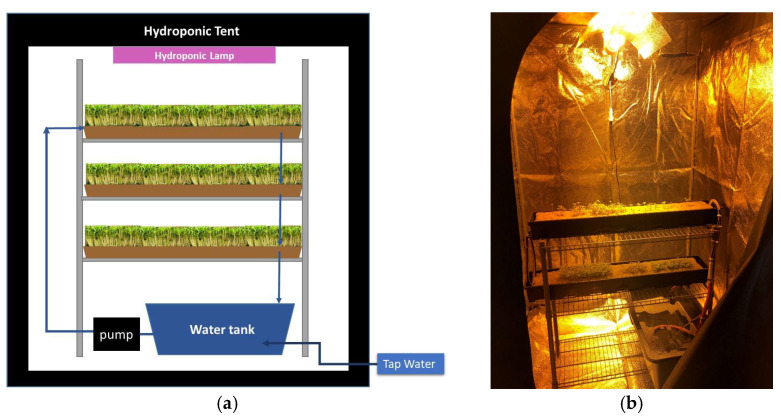
(**a**) Schematic representation and (**b**) photograph of the DIY hydroponic installation.

The authors of this study assembled and placed a wick-type hydroponic installation (Figure 1) in the premises of SCiO P.C. This type of hydroponic installation was chosen due to its low cost, the ease of installation and use, and the accessibility of basic components for online purchase. By using everyday items, anyone can quickly and affordably build their own low-maintenance installation. The shelving unit, hydroponic tent, pump, airstone, hydroponic lamp, and microgreen seedling hydroponic growing mats (biodegradable and compostable jute fiber from coco coir) were ordered online through commercial sites, such as Alibaba (www.alibaba.com) and Amazon (www.amazon.com). The tubes and water tank were purchased at general hardware stores in the area. Seeds for *Ocimum basilicum* (small-leaf basil, hereafter referred to as “small-leaf” for brevity), *Ocimum basilicum* “mammoth” (wide-leaf basil, hereafter referred to as “mammoth” for brevity), *Ocimum basilicum purpurescens* (purple basil, hereafter referred to as “purpurescens” for brevity), and *Brassica oleracea* var. *italica* (broccoli, hereafter referred to as “broccoli” for brevity) were purchased at a local plant store. Simple tap water was used to water the plants without the use of any nutrient powders.

### 2.2. Immersible Photonic Sensors and Reader

The immersible photonic sensors (US Patent 11,119,040; EPO Patent Application: EP3532825A1), their principle of operation, and the accompanying reader configuration have been described in detail in [34]. Briefly, the silicon-based photonic sensors were fabricated at the clean-room facility of the Nanotechnology and Microsystems Laboratory, Institute of Nanoscience and Nanotechnology, NCSR “Demokritos”, on 4″ wafers with a 5 μm thick silicon dioxide bottom cladding layer and an LPCVD-grown silicon nitride layer (SiMat, Kaufering, Germany). The fabrication process followed standard microfabrication techniques, including a lithography step and a dry etching step to define the photonic circuit waveguides, the deposition of a 2 μm thick silicon dioxide top cladding layer followed by a second lithography, and a wet etching step to define the sensing windows for the Mach–Zehnder interferometers (MZIs) over one arm (the other remains covered by the top cladding layer), as schematically depicted in Figure S1a–f and as shown in microscope images in Figure S2b (Supplementary Materials). The completed wafers were then diced into individual chips measuring 25 mm × 2.0 mm (Figure S1g, Supplementary Materials). Each chip contained 2 BB-MZIs of different resonant frequency output spectra because they were originally designed for biochemical determinations [34,35], and one of the interferometers serves as a reference. In this work, the second (reference) BB-MZI was disregarded. As mentioned before, the concept was to record the real-time spectral evolution due to the dynamic adhesion/disociation of molecules from the basil extract onto the sensor-exposed silicon nitride surface; hence, the exposed sensing arms of the BB-MZIs did not undergo any surface treatment apart from cleaning in a prianha solution prior to use.

**Figure 2:**
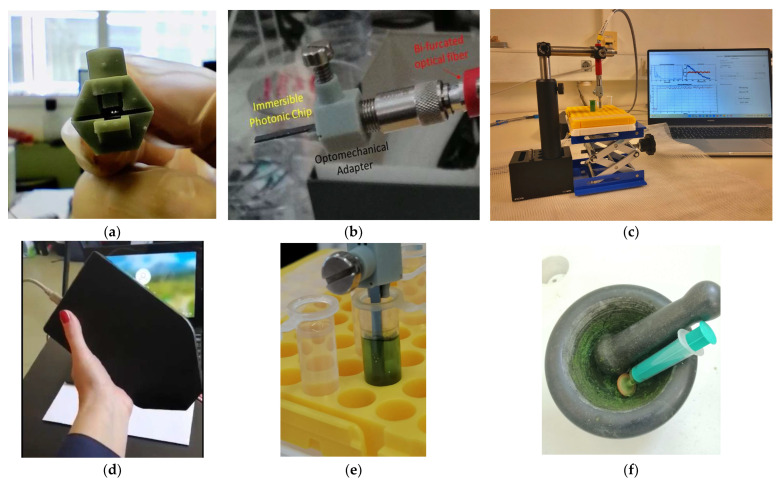
Photographs of (**a**) the front side of the optomechanical adapter (the two cores of the bi-furcated fibers can be seen in the middle) where the immersible photonic chips are inserted; (**b**) the optical adapter with the chips inserted and optomechanically coupled to the bifurcated fiber; (**c**) the experimental setup of the immersible sensors; (**d**) the portable handheld reader; (**e**) close-up photograph showing the immersible sensor during a measurement directly dipped into the basil extract and (**f**) the pestle and mortar preparation of the basil extracts.

The input and output waveguide coupling edges were located on the same short chip sides (Figure S2a,c), while the BB-MZIs were located on the opposite free end of the chip so that they could be directly immersed in any liquid sample (Figure 2c,e and Figure S2b,c, Supplementary Materials), rendering them microfluidics-free. The chips were optomechanically coupled to the light source (high-brightness white LED, Ushio Europe B.V.; Oude Meer, Haarlemmermeer, The Netherlands) and to the recording medium (spectrometer, Flame-T-VIS-NIR, Ocean Insight) through a specially designed adapter (Figure 2a,b) fabricated by 3D printing and a bifurcated optical fiber (Ocean Insight; Duiven, The Netherlands). The light source and the spectrometer were integrated into a portable configuration (Figure 2d) and powered by a laptop through a USB connector. The laptop also included dedicated software for signal acquisition and real-time analysis of the results operated through a simple user interface (Figure 2c).

The photonic sensors were used by simple immersion, similar in use to a strip test, into the basil extracts (Figure 2d) by raising and lowering the sample holder by a small lab jack (Figure 2c). The dynamically evolving spectra were recorded for 50 μs (spectrometer integration time) every 100 μs. The signal processing algorithm was converted in real time through FFT, thus converting the spectral shift into a phase change signal [34] and showing it alongside the spectra to the end user through the dedicated user interface. In this reader configuration (i.e., when employing the particular light source and spectrophotomter model), the noise level is 0.03 rad. Because in the present approach only phase changes due to refractive index changes induced by the plant extracts are measured, the limit of detection (LOD) can be set to 3 times the noise level; in other words, at 0.09 rad.

### 2.3. Microgreen Culitvation

Initially, two types of basil (small-leaf and mammoth) were planted in order to familiarize ourselves with hydroponics, to test for appropriate solvents, and to devise an appropriate yet simple protocol for measuring the plant extracts with the immersible sensors. The purpose was to determine if the plant extracts could be measured accurately using the immersible sensors and to establish familiarity with hydroponics.

Afterwards, all available plants (including all three types of basil and broccoli) were hydroponically cultured and harvested 10 days after seeding. This round of harvesting was used to determine if differentiation among different plants and among plants of the same species could be achieved by the sensors.

Subsequently, basil was chosen as a study case and the edible species of small-leaf and mammoth basil were grown for a total of 3 weeks. The scope of this round was to investigate whether the immersible probes could provide indirect information about the optimum harvest time. Portions of the basil microgreens were harvested at 1-week intervals, and each sample was split in half. One half was immediately transported to the Agricultural University of Athens for measurement using standard analytical techniques to determine the total phenolic content and antioxidant activity, while the other half was measured using the immersible sensor setup. To maintain freshness, the microgreens were stored in a freezer at −20 °C until testing.

### 2.4. Determination of Total Phenolic Content and Antioxidant Activity

Ethanolic extracts were prepared using 2 g of fresh samples diluted in 20 mL ethanol (Sigma-Aldrich, St. Louis, MO, USA). Ultrasound-assisted extraction (UAE) was performed in an ultrasonic bath operating at 35 kHz for 15 min at 25 °C.

#### 2.4.1. Folin–Cioacalteu Assay

To determine the content of total phenolic components, a sodium carbonate (Na_2_CO_3_) (Merck KGaA (Darmstadt, Germany) solution, 20% *w*/*v*, was prepared for the formation of an alkaline environment during the reaction of the sample with Folin–Ciocalteu reagent (Sigma-Aldrich, St. Louis, MO, USA). Then, 1.5 mL H_2_O, a 25 μL sample, and 125 μL Folin–Ciocalteu reagent were mixed, and after 3 min, 375 μL of Na_2_CO_3_ and 475 μL of deionized water were added. After 2 h of incubation in the dark, measurement of absorbance at 725 nm in the ultraviolet spectrometer (visible UV-Vis) (Model V-1200, VWRM Spectrophotometers) followed. All samples were measured three times. The results are expressed in Gallic acid (Sigma-Aldrich, St. Louis, MO, USA) equivalents, for which a standard curve was constructed.

#### 2.4.2. DPPH Assay

The DPPH assay was performed as previously described in [36]. In detail, ethanolic extracts were tested for antioxidant activity with the 2,2-diphenyl-1-picrylhy-drazyl (DPPH^•^) radical assay. The DPPH radical (DPPH^•^) (Sigma-Aldrich, St. Louis, MO, USA) solution (60 μM) was prepared in ethanol. Then, 3.0 mL of the DPPH^•^ solution was mixed with 30 μL of the prepared extract. The solution was then mixed and incubated at room temperature in the dark for 1 h; then, absorbance at 515 nm was recorded. A calibration curve was obtained by using the Trolox (Aldrich, Steinheim, Germany) standard ethanolic solution, and the results were expressed in terms of the Trolox Equivalent Antioxidant Capacity (TEAC) as mg Trolox equivalents per mL. All assays were carried out in triplicates.

#### 2.4.3. ABTS Assay

The DPPH assay was performed as previously described in [36]. In detail, the 2,2′-azino-bis(3-ethylbenzothiazoline-6-sulfonate) radical cation (ABTS^•+^) solution was prepared by the reaction of 7 mM ABTS^•+^ (Sigma-Aldrich, St. Louis, MO, USA) and 2.45 mM potassium persulphate (Merck KGaA, Darmstadt, Germany) after incubation at room temperature in the dark for 12–16 h. The (ABTS^•+^) solution was thus diluted with ethanol to obtain an absorbance of 0.700 ± 0.020 at 734 nm. After the extension of 3.0 mL of diluted (ABTS^•+^) solution (A734 nm = 0.700 ± 0.020) to 30 μL of ethanolic extracts, the absorbance reading at 30 °C, t = 6 min after initial mixing (A sample) was acquired. A calibration curve was obtained using the Trolox (Aldrich, Steinheim, Germany) standard ethanolic solution, and the results were expressed in terms of Trolox Equivalent Antioxidant Capacity (TEAC) as mg Trolox equivalents per mL. All determinations were carried out in triplicates.

## 3. Results and Discussion

### 3.1. Defining the Measuring Protocol

Indirectly determining the nutrient content of plant extracts through dynamic spectral evolution is a novel and unexplored approach. Therefore, it was crucial to establish an optimal method for preparing the plant extracts. As mentioned, the underlying concept was to utilize the immersible photonic sensors as an analysis platform aimed to enable on-site testing with simplified procedures using readily available household utensils and cost-effective chemicals, which is similar to self-test kits. Furthermore, an investigation was conducted to assess the reusability of the photonic sensors by examining their feasibility for rinsing and subsequent measurement of multiple samples. A review of the literature on sample preparation for plant studies revealed the prevalent use of acetone and alcohols (methanol or ethanol) in conjunction with fresh plant samples [37,38,39,40,41]. Acetone and ethanol were chosen as solvents to be tested, and a straightforward sample preparation method was devised. This method involved homogenizing the collected samples in the respective solvent with the aid of a standard pestle and mortar, transferring the resulting pulp into a syringe, and obtaining the “extract” by filtration through a 400 μm pore filter (Figure 2e,f). The plant extracts as well as the various solvents used to test the rinsing process were placed in 5 mL Eppendorf tubes placed in a suitable holder (Figure 2c,e). This study was performed with mammoth and small-leaf basils harvested by the first seeding of the hydroponic installation (see Section 2.3).

Acetone was studied first based on the findings of [37]. Based on the literature search, 1 gr of each basil plant was mashed in 3 mL of acetone in the mortar, and the following procedure was followed:

Step 1: The sensor was dipped into pure acetone to obtain a baseline.

Step 2: Then, the sensor was dipped into the basil extract for 1 min.

Step 3: The chip was then rinsed for at least 2 min in fresh acetone to see whether the chip surface could be properly rinsed and whether the signal would return to the baseline levels.

Steps 1–3 were repeated to verify whether rinsing was adequate and whether the chip could be sequentially used for multiple measurements.

**Figure 3:**
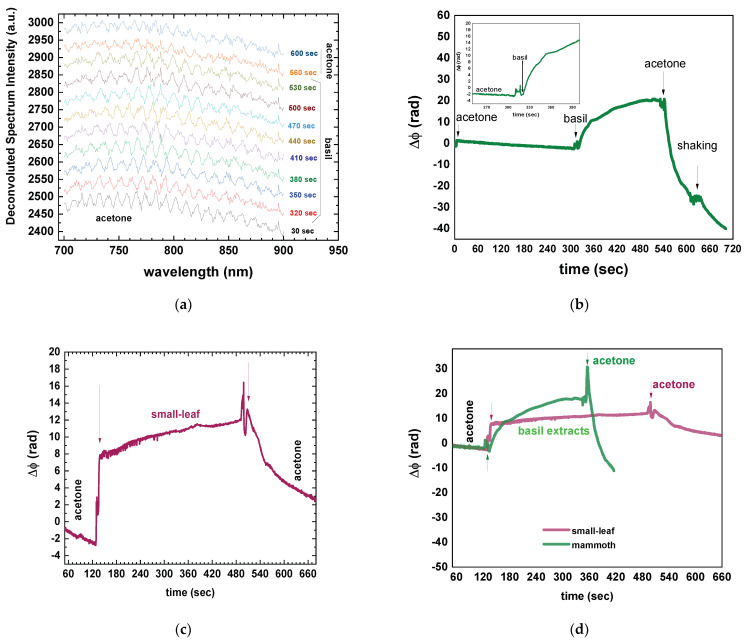

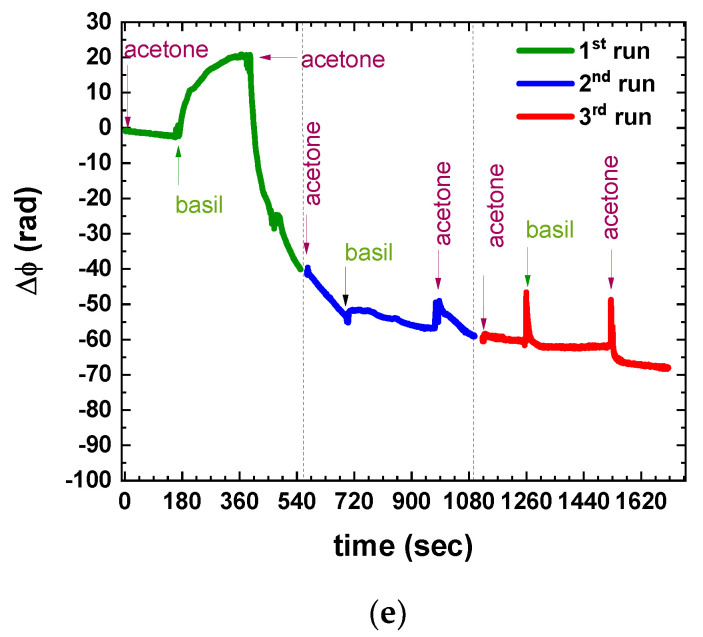
(**a**) De-convoluted spectra (to the LED shape) of the first mammoth basil measurement with their time stamps indicated. The spectra have been vertically shifted for clarity. The 3.5 period shift is not evident and can only be observed when analyzed as a phase change in (**b**). (**b**) Phase change signal from the mammoth basil measurement. (**c**) Phase change signal from the small-leaf basil measurement. (**d**) Comparison of the phase change signals for the small-leaf (purple line) and mammoth (green line) basil. (**e**) Phase change signal testing the effectiveness of acetone as a rinsing agent demonstrating three consecutive measurements with the same chip and the same mammoth extract. Arrows in (**b**–**e**) indicate when the sensor was raised and dipped in the next solution.

Figure 2 presents the initial measurement of mammoth basil leaf extract, capturing shifting spectra (Figure 3a) and temporal phase changes (Figure 3b). Notably, a substantial phase shift of approximately 20 rad (~3.5 cycles) occurred, with the most significant change transpiring within the initial 30 s (inset Figure 3b). Comparatively, the phase change signal exhibited distinct characteristics when measuring small-leaf basil extract (Figure 3c) in comparison to mammoth basil (Figure 3d). The former displayed a steeper, almost step-like gradient, while the latter exhibited a smoother transition, requiring over 60 s to reach a plateau. These observations, consistent across two different chips for each basil type, indicated that monitoring the phase change and its slope sufficed to distinguish between the plants (Figure 3d), rendering the complete spectral evolution unnecessary. Consequently, subsequent investigations focused exclusively on analyzing the phase changes.

Nevertheless, attempts to rinse the photonic chip for reusability revealed an intriguing finding. The phase signal failed to return to its original values and displayed a persistent shift (Figure 3d), indicating that the removal of material from the Eppendorf walls resulted in a continuous alteration of the solvent’s refractive index. Furthermore, subsequent immersion of the sensor in basil extract for consecutive measurements yielded varied, reduced phase change values, highlighting acetone’s inability to completely eliminate adhered molecules from the sensor surface. Essentially, an adlayer had formed on the sensor surface, which was impervious to complete removal. Subsequent “rinsing” led to the re-establishment of additional molecules on the sensor surface, causing the layer to thicken and resulting in an almost plateau-like phase signal. In essence, the adlayer reached a thickness relative to the evanescent field of the propagating light, rendering it perceptible as the upper cladding layer. This observation also suggested that the chosen concentration for the plant pulps might have been excessive and that it should be reduced, thus leading to less dense extracts.

Building upon the previous findings, the suitability of ethanol as a solvent was investigated, accompanied by a reduction in plant concentration to 1 g per 5 mL of solvent. The potential formation of adlayers on the sensor surface prompted not only plant concentration reduction, but also the implementation of a more rigorous rinsing protocol, akin to regeneration methods employed by the authors in biodetection schemes. Consequently, the measurement steps involved sequential treatment with ethanol (to establish a baseline), plant extract, ethanol (for rinsing), DI water (to remove ethanol residue), 100 mM HCl in DI water (to eliminate any adlayers), DI water (for acid rinse), and ethanol (to ensure complete rinsing and to restore baseline). Multiple cycles of these measurements were performed to assess chip cleanliness, reusability, and consistency of results, thereby determining the extent of sustainable reusability. This study was performed with all four plants (small-leaf, mammoth, purpurescens, and broccoli) harvested 10 days after seeding from the DIY hydroponic installation.

**Figure 4:**
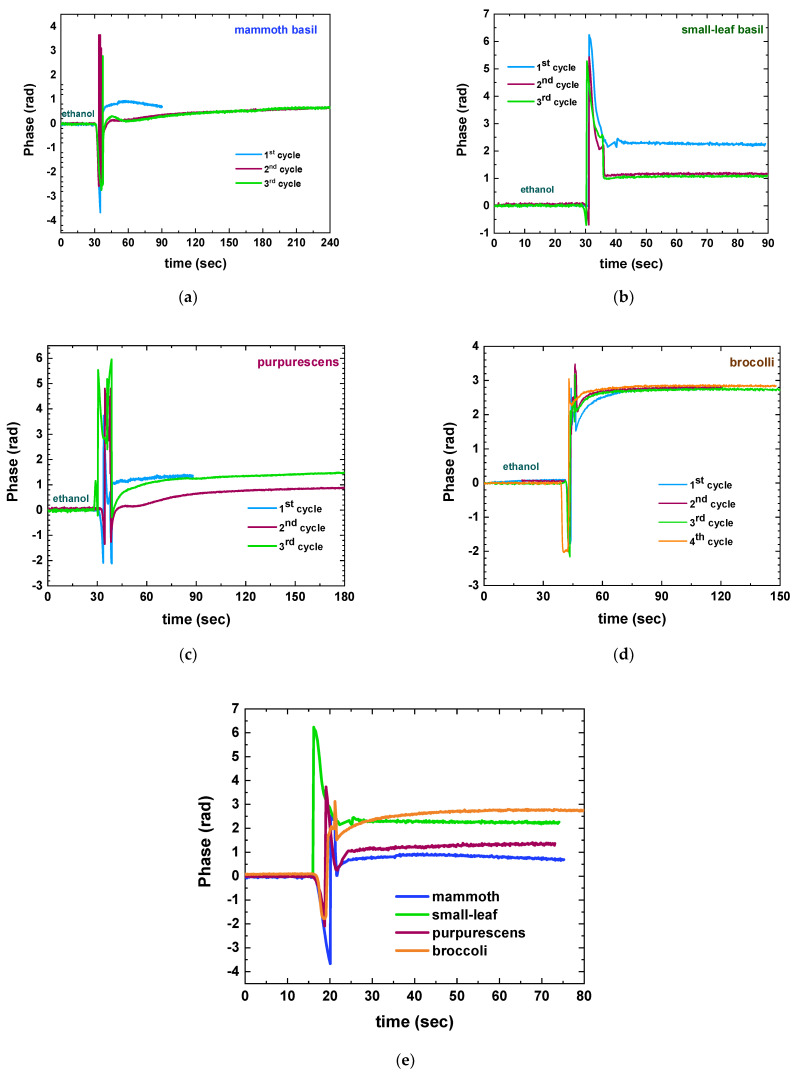
Phase signal changes of ethanol-to-plant extract immersion after repeating cycles of dipping and rinsing for (**a**) mammoth basil, (**b**) small-leaf basil, (**c**) purprescens basil, and (**d**) broccoli. (**e**) Comparison of the phase change signals from each plant after the first dipping of a pristine photonic sensor (mammoth: blue line; small-leaf: green line; purpursescns: magenta line; broccoli: orange line).

Figure S3 (Supplementary Materials) presents the comprehensive sequential experiments conducted to simultaneously assess the suitability of ethanol as a solvent and the repeatability of measurements. The consolidated findings are summarized in Figure 4, where the transitions from ethanol to plant extract in repeated cycles are plotted for all plants. Notably, ethanol established a reliable baseline with no observed shifts, distinguishing it from acetone. However, among the tested plants, repeatable results were achieved up to four rinsing cycles only for broccoli (Figure 4d) and up to two cycles for purpurescens (Figure 4c), while both mammoth and small-leaf basil failed to deliver consistent results beyond the initial rinsing step (Figure 4a,b, respectively). The most important result, though, lies in the distinct phase signals exhibited by the four plants, acquired from the initial immersion (first cycle) of “fresh” immersible sensors, characterized by unique slopes indicative of each plant (Figure 4e). This outcome supports the original hypothesis that the proposed approach has the capability to differentiate between different plants, even within the same species.

**Figure 5:**
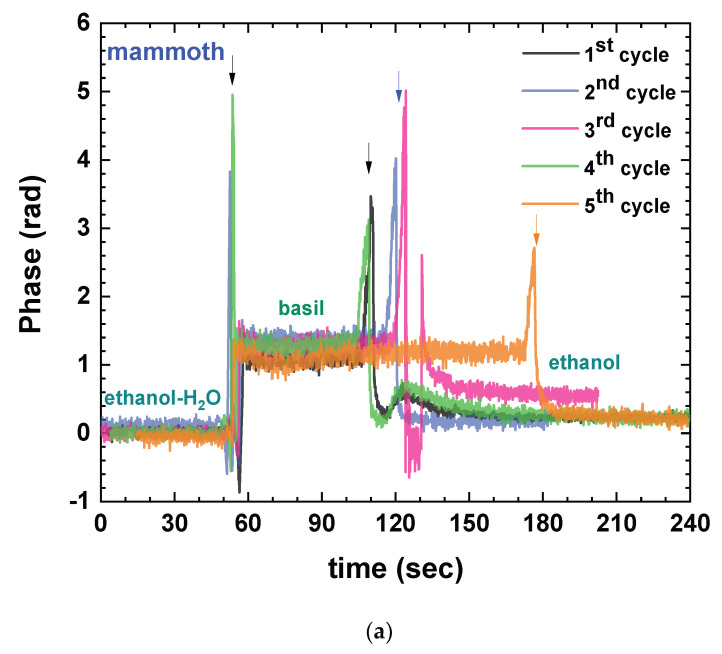

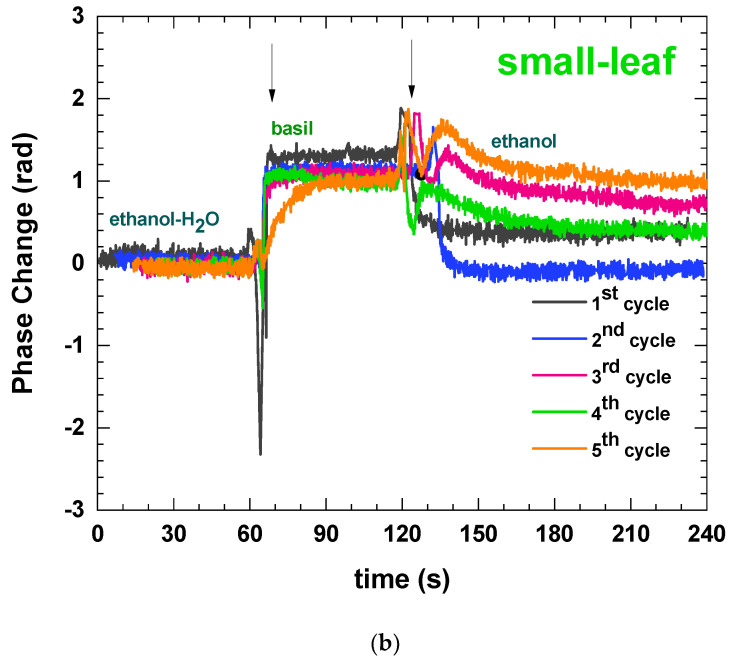
Consolidated phase change signals from 5 repeated cycles of ethanol–water/basil extract/ethanol for (**a**) mammoth and (**b**) small-leaf basil. Arrows indicate the transition from one liquid sample to the next.

Upon closer examination of the signals in Figure S3, it was suggested that the additional step of rinsing with water and hydrochloric acid may not be necessary. Furthermore, during the measurements, it was observed that ethanol evaporated rapidly and needed to be replenished multiple times. In line with the objective of maintaining a simple measurement protocol with minimal materials, the authors adopted a simplified approach. A mixture of 30% *v*/*v* DI water and 70% *v*/*v* ethanol was utilized as a baseline and as the solvent for mashing the plant, followed by immersing the sensor in the plant extracts and rinsing with pure ethanol until the signal stabilized. This simplified approach was tested with 2-week-old microgreens of mammoth and small-leaf basil. As depicted in Figure 5, this simplified approach surprisingly yielded repeatable signals for up to four testing–rinsing cycles. This approach was finally chosen to perform the final correlation experiments.

### 3.2. Correlation Experiments

**Figure 6:**
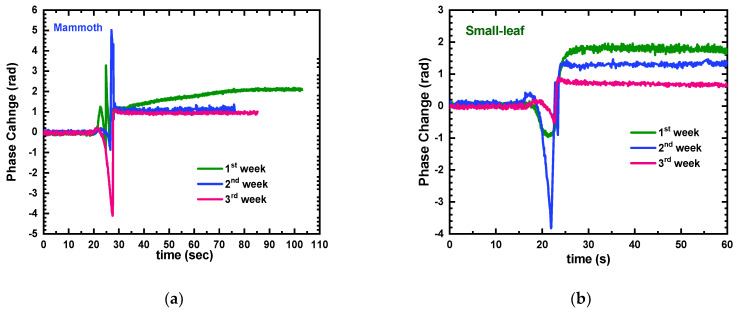
Weekly phase change signals for (**a**) mammoth and (**b**) small-leaf basil over a period of 3 weeks (1st week: green line; 2nd week: blue line; 3rd week: magenta line).

**Figure 7:**
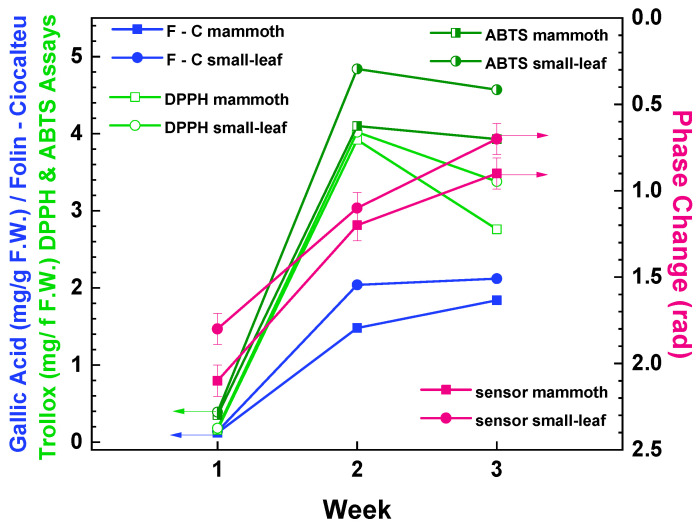
Total phenolic content expressed in Gallic acid concentration (determined by the Folin–Ciocalteu assay: blue lines, left axis) and antioxidant activity expressed in Trolox concentration (determined by the DPPH and ABTS assays: green lines, left axis; DPPH: open symbols; ABTS: half-solid symbols) in conjunction with the photonic sensor signals (phase change) expressed in rads (magenta lines; right axis. The y-axis has been reversed for clarity). Errors bars are set to ±3SD. Mammoth basil: squares; small-leaf-basil: circles.

The final correlation experiments involved the hydroponic cultivation of two edible basil species: specifically, mammoth and small-leaf basil microgreens. Over a period of 3 weeks, with weekly harvesting, the plants were grown as outlined previously. Measurements were conducted using immersible sensors on half of the plants, while the remaining half was directly transferred to the Agricultural University of Athens for analysis of their total phenolic content and antioxidant activity (see Section 2.4 for details). The summarized results from all methods are presented in Table 1, and the phase signal measurements obtained from the immersible photonic sensors are showcased in Figure 6. Figure 7 provides a summary of the measurements from all methods, including the analytical techniques and the immersible photonic sensors.

To determine the antioxidant activity of mammoth and small-leaf basil varieties, two assays were conducted: the DPPH assay, suitable for lipophilic antioxidants, and the ABTS assay, more suitable for hydrophilic ones. Table 1 indicates that basil samples harvested in week two exhibited higher antioxidant activity in both DPPH and ABTS assays. In the DPPH assay, mammoth and small-leaf samples harvested in week two displayed the highest concentrations of 3.92 mg Trolox/g F.W and 4.1 mg Trolox/g F.W, respectively. Similarly, in the ABTS assay, both basil types harvested in week two demonstrated higher antioxidant activity, with concentrations of 4.02 mg Trolox/g F.W and 4.82 mg Trolox/g F.W for mammoth and small-leaf samples, respectively. Additionally, the Folin–Ciocalteu assay was performed to determine the total phenolic content. Notably, samples harvested in week three exhibited higher content compared to other weeks. Specifically, the mammoth basil sample displayed a concentration of 1.84 mg Gallic acid/g F.W, while the small-leaf basil sample showed 2.12 mg Gallic acid/g F.W. According to these standard measurements, the second week is the optimal time to harvest basil microgreens for maximal nutritional value, as samples showcased higher antioxidant activity and an increase in total phenolic content.

Turning our attention to the photonic sensor results, the phase change signal obtained from the basil extracts notably demonstrates a gradual decrease over the course of the weeks (note that the axis in Figure 6 has been reversed to better illustrate this trend). Interestingly, this downward trend aligns with the findings from the Folin–Ciocalteu assay, hinting at a potential link between the sensor signal and the total phenolic content of basil microgreens. However, it is important to note that this study was limited in breadth, and that further investigations are required to validate this hypothesis, as was explained in the recent report by Tran et al. [30] that employed spectral multi-channel spectral sensors as plant reflectance measuring devices to determine the total phenolic content of basil. A more comprehensive exploration would involve a larger set of experiments conducted across multiple generations of hydroponically cultivated crops. Moreover, a more detailed analysis could involve examining the signal gradients and scrutinizing the rate at which the signal approaches a plateau, providing stronger evidence for a potential correlation between the nutrient contents of microgreens and the sensor signals. In addition, it would be useful to determine through other methods the content of chromophore molecules within the various generations of the plants because these could affect the refractive index of the plant extracts. Advanced techniques, such as machine learning algorithms, could enhance this detailed analysis, provided a substantial number of experiments are performed over several generations of hydroponically cultivated crops.

## 4. Conclusions

In conclusion, the concept of using immersible photonic sensors to assess the nutrient content of hydroponically cultivated plants has shown promising results. The initial experimentation involved testing various microgreen pulps, which established the varying gradient of phase changes between different plant species and within plants of the same species. Building upon this foundation, this study focused on two types of basil microgreens, monitoring them at 1-week intervals over a 3-week period, and comparing the sensor signals with the total phenolic content and antioxidant activity determined by Folin–Ciocalteu, DPPH, and ABTS assays.

The results obtained from the immersible photonic sensors hinted at a possible correlation between the detected signals and the total phenolic content of the microgreens. Specifically, it was observed that the signals exhibited a consistent trend that aligned with the changing nutrient composition of the plants. This finding suggests that the immersible photonic sensors have the potential to serve as a tool for assessing the nutrient content of hydroponically grown plants.

While these findings are preliminary and serve as proof of concept, they hold significant implications. They indicate that the optimal harvest time for hydroponically farmed microgreens lies around the 14th day (2 weeks after planting). This knowledge could be instrumental in maximizing the nutritional value of these crops.

Furthermore, these novel photonic sensors offer several advantages, including the potential for on-site, rapid, cost-effective, and efficient assessment of plant nutrient content. If further research and development support these initial findings, it could lead to the creation of a valuable tool that empowers farmers and cultivators to determine the optimal harvest time for their hydroponically farmed plants.

In summary, the application of immersible photonic sensors in conjunction with standard analytical techniques has demonstrated a correlation between the sensor signals and the total phenolic content of basil microgreens. This represents a promising step toward developing a practical and accessible method for assessing plant nutrient content. Future investigations, including expanded experiments and the integration of advanced analysis techniques, such as machine learning algorithms, will be vital for consolidating these initial findings and unlocking the full potential of this innovative technology in the field of hydroponics.

## Figures and Tables

**Table 1:** Results from the Folin–Ciocalteu, DPPH, and ABTS assays in conjunction with the measurements from the immersible photonic sensors.

Basil Type	Week	Folin–Ciocalteu Assay (mg Gallic Acid/g F.W.)	DPPH Assay (mg Trolox/g F.W.)	ABTS Assay (mg Trolox/g F.W.)	Immersible Sensors Phase Change (Rad)
mammoth	1	0.12	0.16	0.35	2.1
	2	1.48	3.92	4.1	1
	3	1.84	2.76	3.93	0.9
small-leaf	1	0.14	0.18	0.39	1.8
	2	2.04	4.02	4.84	1.3
	3	2.12	3.38	4.57	0.7

## Data Availability

The data presented in this study are available upon request from the corresponding author.

## Supplementary Materials

The following supporting information can be downloaded at: https://www.mdpi.com/article/10.3390/s23135937/s1, Figure S1: Schematic representation of the immersible photonic sensor fabrication steps at the area of the PIC where one of the BB-MZIs is located (cross-sectional view): (a) starting wafers with 5 μm SiO_2_ cladding layer and LPCVD Si3N4 waveguide core layer, (b) 1st lithographic step and (c) dry etching for the definition of the waveguides and the photonic circuit, (d) SiO_2_ top cladding depo-sition, (e) 2nd lithographic step for the removal of the cladding layer from the BB-MZI sensing arm, and (f) wet etching and sensing window definition. (g) Photograph of the immersible chips after dicing. Figure S2: (a) Three-dimensional schematic rendering (not in scale) of the immersible photonic chip and the in- and out-coupling to the bi-furcated optical fiber. The optomechanical adapter is not shown in the rendering for clarity. Optical microscope images of (b) the BB-MZI area after the opening of the sensing windows (on the left, one can discern part of the first BB-MZI of the shorter sensing arm and on the right, the second BB-MZI with the longer sensing arm as described in [25]), and (c) the short ship side (before dicing) showing the input and output waveguides. Figure S3: Repeating cycles of testing and rinsing with pure ethanol sued as the solvent and baseline for (a) mammoth basil, (b) small-leaf basil, (c) purple basil and (d) broccoli.
