# Supplementary material for: Determining the Nutrient Content of Hydroponically-Cultivated Microgreens with Immersible Silicon Photonic Sensors: A Preliminary Feasibility Study
Source: Sensors (Basel). 2023 Jun 26;23(13):5937. doi: 10.3390/s23135937 (PMC10346951; doi:10.3390/s23135937)
Supplement: Supplementary file 1 [file sensors-23-05937-s001.zip › sensors-2456705-supplementary.pdf]

sensors

MDPI

SUPPLEMENTARY MATERIALS FOR MANUSCRIPT

Determining the Nutrient Content of Hydroponically-Cultivated Microgreens with Immersible Silicon Photonic Sensors: A Preliminary Feasibility Study

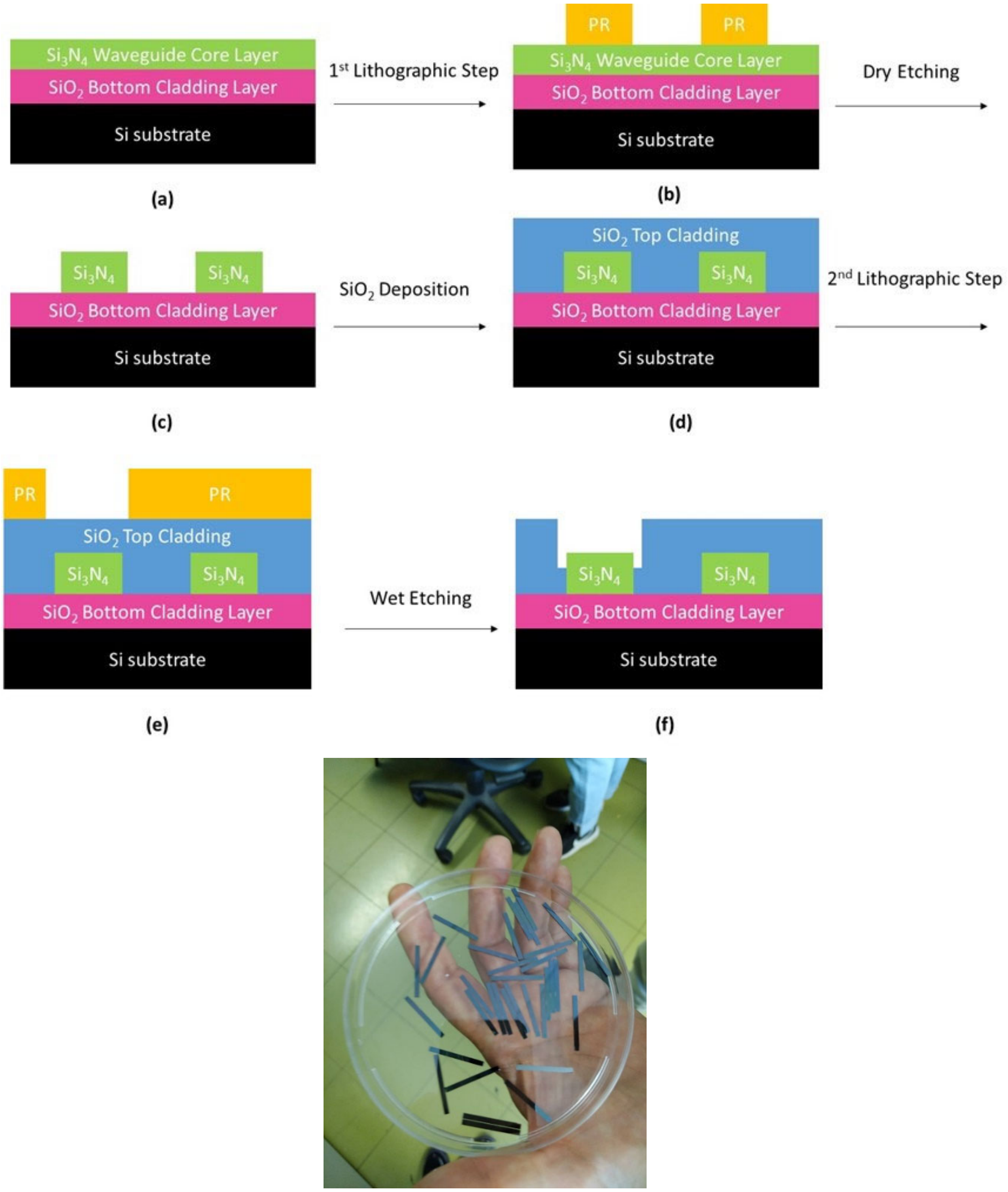

(g)

Figure S1. Schematic representation of the immersible photonic sensor fabrication steps at the area of the PIC where one of the BB-MZIs is located (cross-sectional view): (**a**) starting wafers with 5 μm

SiO_2 cladding layer and LPCVD Si_3N_4 waveguide core layer, (**b**) 1st lithographic step and (**c**) dry etching for the definition of the waveguides and the photonic circuit, (**d**) SiO_2 top cladding deposition, (**e**) 2nd lithographic step for the removal of the cladding layer from the BB-MZI sensing arm, and (**f**) wet etching and sensing window definition. (**g**) Photograph of the immersible chips after dicing.

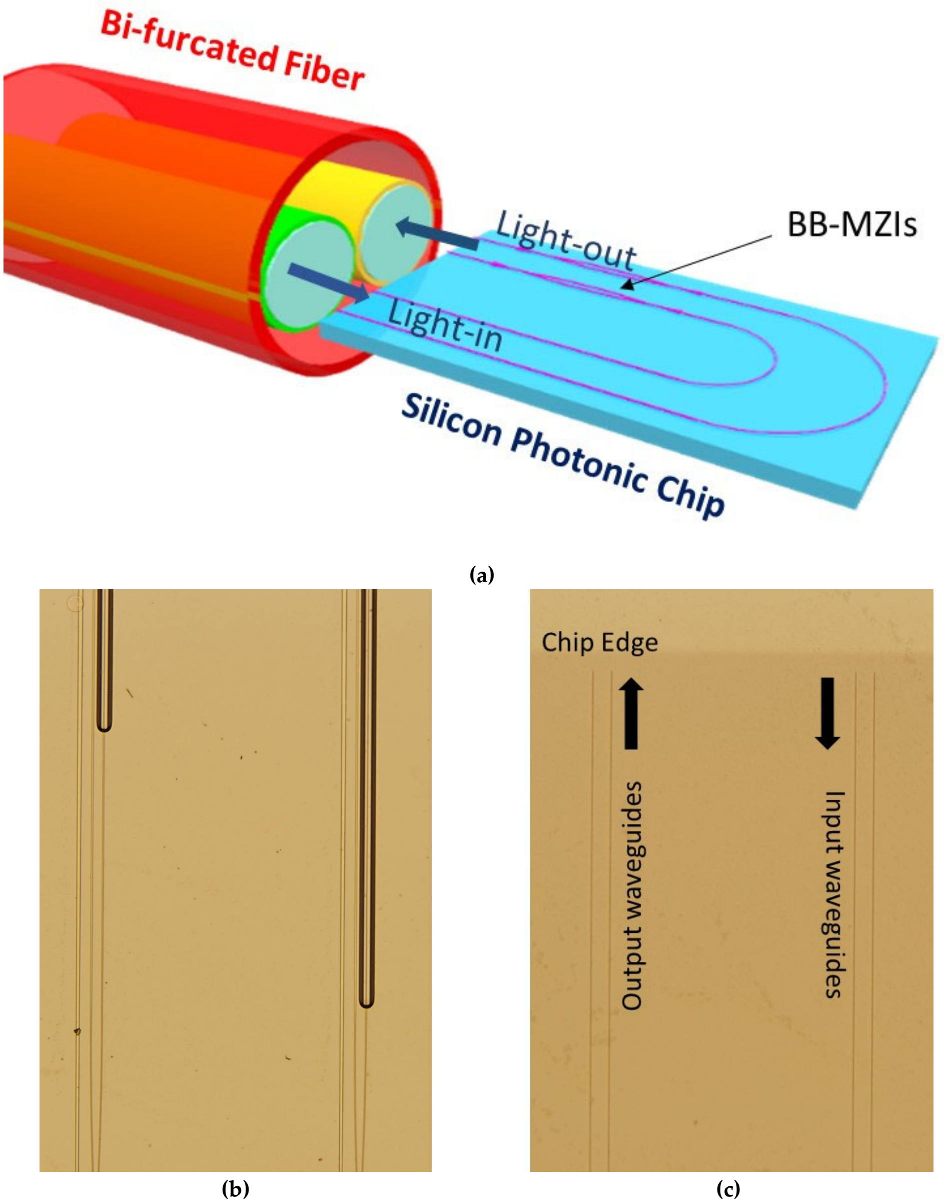

Figure S2. (a) Three-dimensional schematic rendering (not in scale) of the immersible photonic chip and the in- and out-coupling to the bi-furcated optical fiber. The optomechanical adapter is not shown in the rendering for clarity. Optical microscope images of **(b)** the BB-MZI area after the opening of the sensing windows (on the left, one can discern part of the first BB-MZI of the shorter sensing arm and on the right, the second BB-MZI with the longer sensing arm as described in [25]), and **(c)** the short ship side (before dicing) showing the input and output waveguides.

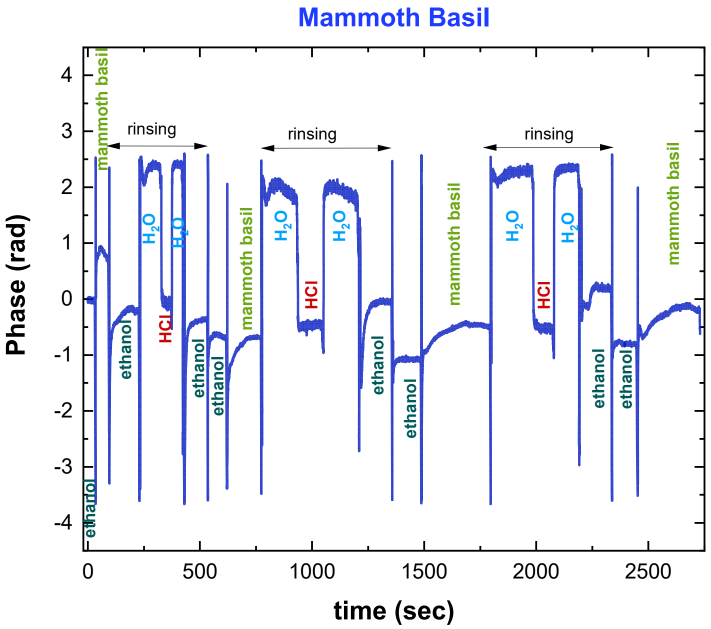
Mammoth Basil
Phase (rad)
time (sec)
rinsing
rinsing
rinsing
mammoth basil
mammoth basil
mammoth basil
mammoth basil
ethanol
ethanol
ethanol
ethanol
ethanol
ethanol
ethanol
ethanol
ethanol
H_2O
H_2O
H_2O
H_2O
H_2O
H_2O
HCl
HCl
HCl

(**a**)

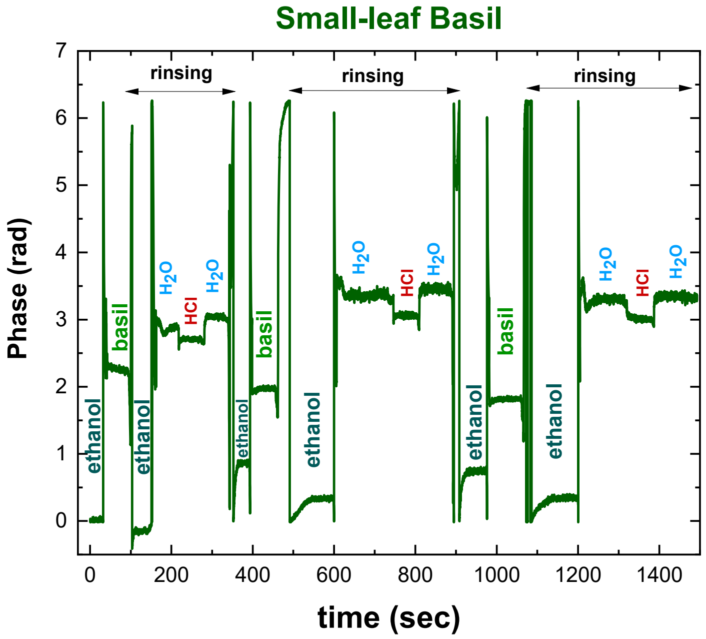
Small-leaf Basil
Phase (rad)
time (sec)
rinsing
rinsing
rinsing
basil
basil
basil
ethanol
ethanol
ethanol
ethanol
ethanol
ethanol
H_2O
H_2O
H_2O
H_2O
H_2O
H_2O
HCl
HCl
HCl

(**b**)

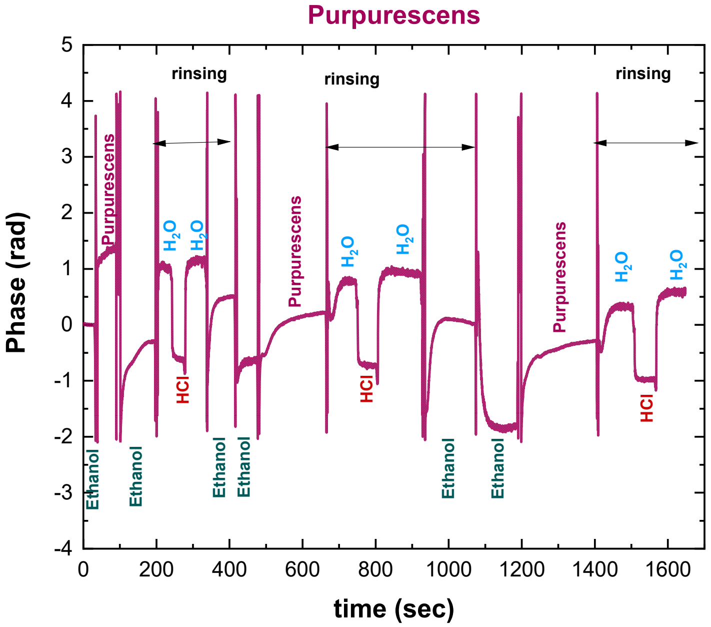

(**c**)

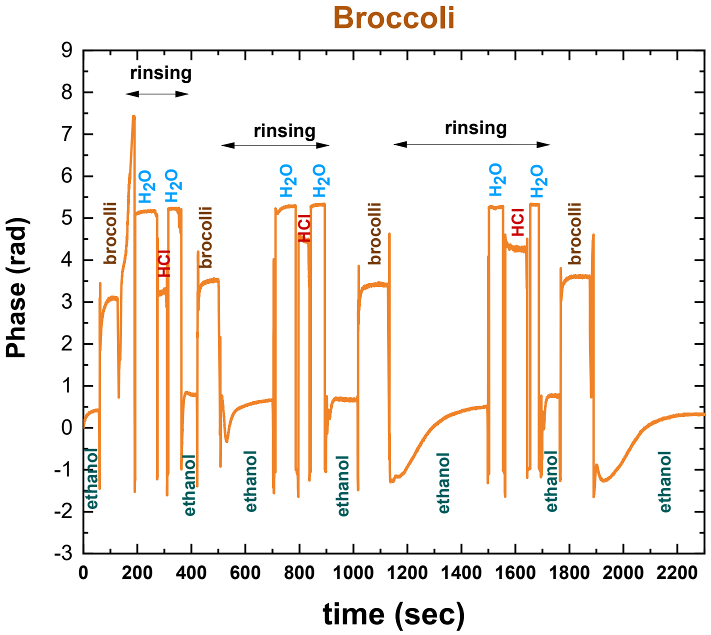

(**d**)

Figure S3. Repeating cycles of testing and rinsing with pure ethanol sued as the solvent and baseline for (**a**) mammoth basil, (**b**) small-leaf basil, (**c**) purple basil and (**d**) broccoli.
